# Compressive Strength of Iliac Bone ECM Is Not Reduced in Osteogenesis Imperfecta and Increases With Mineralization

**DOI:** 10.1002/jbmr.4286

**Published:** 2021-04-04

**Authors:** Michael Indermaur, Daniele Casari, Tatiana Kochetkova, Cinzia Peruzzi, Elizabeth Zimmermann, Frank Rauch, Bettina Willie, Johann Michler, Jakob Schwiedrzik, Philippe Zysset

**Affiliations:** ^1^ ARTORG Center for Biomedical Engineering Research University of Bern Bern Switzerland; ^2^ Swiss Federal Laboratories for Material Science and Technology, Empa Thun Switzerland; ^3^ Shriners Hospital for Children Montreal Canada; ^4^ McGill University Montreal Canada

**Keywords:** OSTEOGENESIS IMPERFECTA, MICROPILLAR COMPRESSION, NANOINDENTATION, MINERALIZATION, BIOMECHANICS

## Abstract

Osteogenesis imperfecta (OI) is an inheritable, genetic, and collagen‐related disorder leading to an increase in bone fragility, but the origin of its “brittle behavior” is unclear. Because of its complex hierarchical structure, bone behaves differently at various length scales. This study aims to compare mechanical properties of human OI bone with healthy control bone at the extracellular matrix (ECM) level and to quantify the influence of the degree of mineralization. Degree of mineralization and mechanical properties were analyzed under dry conditions in 12 fixed and embedded transiliac crest biopsies (control *n* = 6, OI type I *n* = 3, OI type IV *n* = 2, and OI type III *n* = 1). Mean degree of mineralization was measured by microcomputed tomography at the biopsy level and the mineral‐to‐matrix ratio was assessed by Raman spectroscopy at the ECM level. Both methods revealed that the degree of mineralization is higher for OI bone compared with healthy control. Micropillar compression is a novel technique for quantifying post‐yield properties of bone at the ECM level. Micropillars (d = 5 μm, h = 10 μm) were fabricated using focused ion beam milling and quasi‐statically compressed to capture key post‐yield properties such as ultimate strength. The qualitative inspection of the stress–strain curves showed that both OI and healthy control bone have a ductile response at the ECM level. The quantitative results showed that compressive strength is not reduced in OI bone and is increasing with OI severity. Nanoindentation measurements revealed that OI bone tends to have a higher Young's modulus, hardness, and dissipated energy compared with healthy bone. Micropillar strength and indentation modulus increased linearly and significantly (*p* < .0001) with mineral‐to‐matrix ratio. In conclusion, this study indicates that compressive mechanical properties of dry OI bone at the iliac crest are not inferior to healthy control at the ECM level and increase with mineralization. © 2021 The Authors. *Journal of Bone and Mineral Research* published by Wiley Periodicals LLC on behalf of American Society for Bone and Mineral Research (ASBMR).

## Introduction

Osteogenesis imperfecta (OI), also known as brittle bone disease, is a rare genetic disorder. In most cases, OI is caused by mutations in genes encoding type I collagen (COL1A1 and COL1A2), leading to increased bone fragility. These OI types can be categorized according to disease severity into type I (mild), type II (perinatally lethal), type III (severe), and type IV (moderate).^(^ ^1^ ^)^ Individuals with OI have high rates of bone fracture, especially during growth,^(^ ^2^ ^)^ as well as an abnormal multiscale bone structure and composition. Going forward, there is a need to understand the relationship between the mechanical behavior and structure in bone from individuals with OI. In particular, with the increasing use of high‐resolution quantitative peripheral computed tomography (HR‐pQCT) to assess bone structure and strength in clinical trials, bone material property measurements from the OI population are needed. The material properties are used as inputs in computational models for predicting bone failure loads and stiffness to assess the progression of the disease/disorder or the efficacy of treatments.

In general, areal bone mineral density (aBMD) by dual‐energy X‐ray absorptiometry (DXA) and trabecular volumetric bone mineral density (vBMD) by HR‐pQCT in OI individuals are reduced compared with healthy controls.^(^ ^3^, ^4^ ^)^ At the macroscopic scale of transiliac crest biopsies evaluated by histomorphometry, OI cortical bone is thinner and more porous, while OI cancellous bone has fewer trabeculae.^(^ ^5^ ^)^ OI bone has been reported to have a higher degree of mineralization quantified with different methods (eg, microcomputed tomography [μCT],^(^ ^6^, ^7^ ^)^ Raman spectroscopy,^(^ ^7^ ^)^ or quantitative backscattered electron imaging [qBEI)]^8^, ^9^ ^)^). Since the 1990s, bisphosphonates (eg, zoledronate, pamidronate), which inhibit bone resorption, are widely administered to children with OI until growth has ended and are used to a lesser extent in adults.^(^ ^10^, ^11^ ^)^ Bisphosphonates reduce but do not eliminate the incidence of bone fractures,^(^ ^12^, ^13^, ^14^ ^)^ It has been reported that bisphosphonate treatment does not alter the mineralization and the indentation properties at the extracellular matrix (ECM) level in pediatric patients.^(^ ^8^ ^)^

Bone mechanical properties can be classified into elastic and post‐yield (or post‐elastic) regimes. Young's modulus describes elasticity or how much stress is required for the reversible stretching of bonds within the bone. The post‐elastic regime describes the initiation and progression of the breakage of bonds beyond a certain yield strain and is characterized by the maximum (or ultimate) stress sustained by the bone but also by the amount of strain and energy needed to grow a major crack and complete failure. Brittle materials generally have small yield strains, whereas tough materials are able to dissipate large amounts of energy before failure. Bone mechanical behavior in biopsies from individuals with OI has been measured with nanoindentation,^(^ ^15^ ^)^ which is an established technique to characterize the elastic properties but also (post‐elastic) hardness of bone.^(^ ^16^, ^17^, ^18^ ^)^ Nanoindentation studies on iliac crest biopsies show that OI human bone at the ECM level has greater Young's modulus and hardness than healthy controls,^(^ ^8^ ^)^ with one study reporting the contrary.^(^ ^7^ ^)^ Nanoindentation of bone from fracture sites found a reduced Young's modulus and hardness in OI type III compared with OI type I,^(^ ^19^ ^)^ with similar properties between OI types III and IV.^(^ ^20^ ^)^ Although nanoindentation provides relevant properties of bone ECM, this technique does not allow the proper assessment of the post‐yield mechanical behavior because hardness is not a true bulk mechanical property. Moreover, nanoindentation results in a complex stress distribution below the tip, which makes the analysis of the post‐yield behavior close to impossible, especially in a heterogeneous and anisotropic material such as bone. Notably, the post‐yield response at the ECM level is necessary to understand the origin of “brittleness” in OI bone at the macroscopic scale.

A well‐established material science‐based technique to capture the post‐yield properties at the microscale in a more straightforward way is micropillar compression.^(^ ^21^, ^22^, ^23^ ^)^ This method was recently applied to dried and hydrated ovine tibias.^(^ ^24^, ^25^ ^)^ Bone micropillars (geometry: 5 μm in diameter and 10 μm in height) were shaped using focused ion beam (FIB) milling in the axial and transversal direction of osteons and then quasi‐statically compressed to failure. The post‐yield ductile behavior at the ECM level was anisotropic with strain softening in axial pillars and strain hardening in transverse pillars.

**Fig 1:**
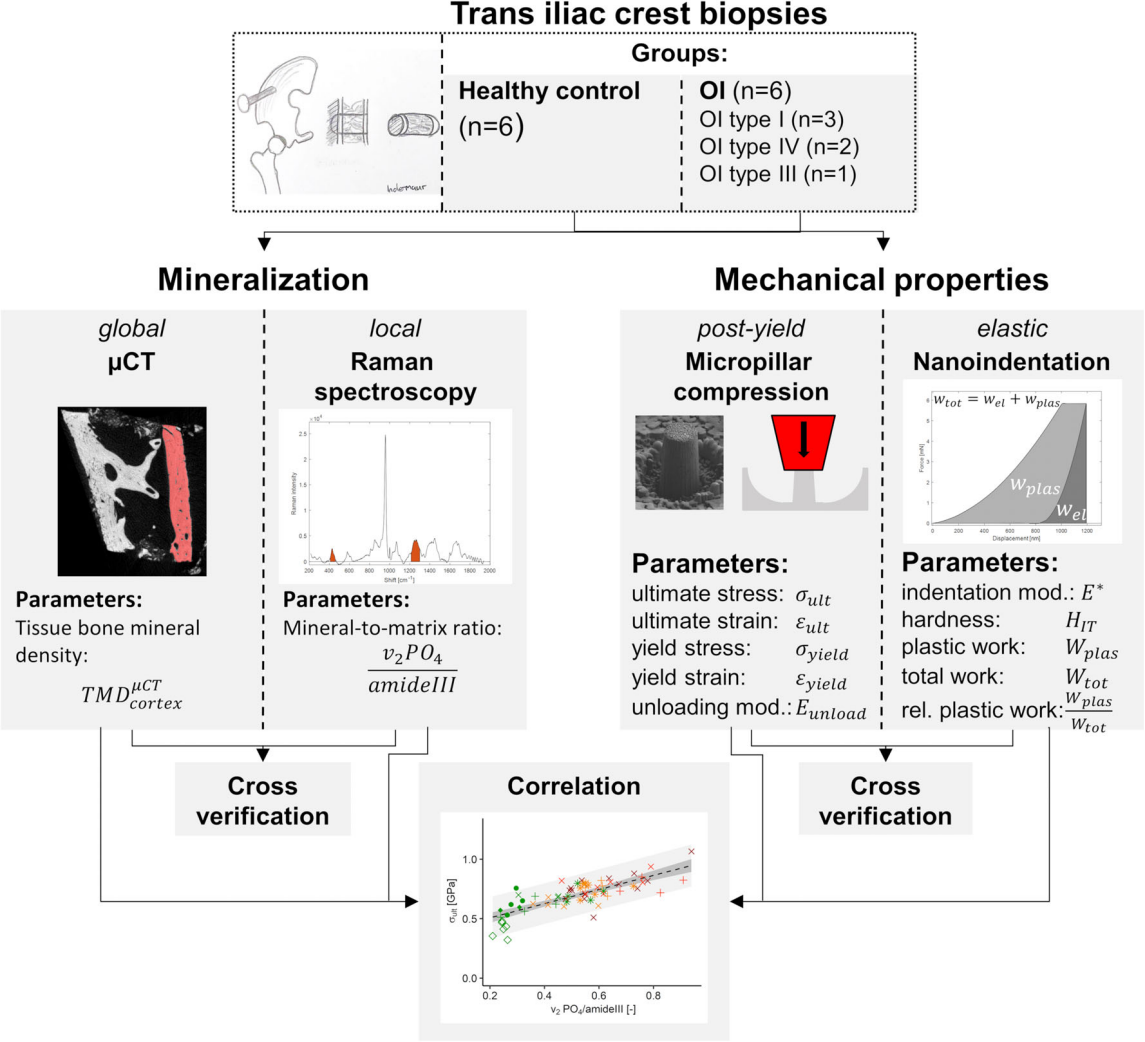
Illustration of the study pipeline. The degree of mineralization (μCT and Raman spectroscopy) and major mechanical properties (micropillar compression and nanoindentation) were both measured with two methods on 12 iliac crest biopsies (healthy control = 6; OI type I *n* = 3; OI type IV *n* = 2; and OI type III *n* = 1). Mineralization and mechanical properties were cross‐verified and mechanical properties were correlated with the local degree of mineralization.

Bones resist deformation and fracture through their multiscale structure. The differences in bone structure and composition in OI compared with healthy bone and their relation to mechanical behavior are complex. Micropillar compression tests directly probe the mechanical behavior of bone at the ECM level and have the potential to reveal differences in elastic and post‐yield mechanical behavior of OI versus control transiliac crest bone biopsies. We hypothesize that (i) the compressive mechanical properties at the ECM level are inferior in OI bone than in healthy bone, and (ii) elastic modulus, yield, and ultimate strength but also brittleness of bone ECM increase with the degree of mineralization. We support the micropillar compression test with nanoindentation for comparison to reported studies and measurements of the degree of mineralization with μCT and Raman spectroscopy (Fig. 1).

## Materials and Methods

### Biopsies

This study comprises 12 transiliac crest biopsies (healthy control *n* = 6, OI type I *n* = 3, OI type IV *n* = 2, and OI type III *n* = 1). OI and healthy control biopsies were obtained from two distinct previous studies that applied the same extraction, fixation, embedding, and conservation protocols.^(^ ^26^, ^27^ ^)^ A detailed overview of the samples is listed in Table 1. Five of 6 OI patients received a bisphosphonate treatment (pamidronate [PAM]) for approximately 5 years.

**Table 1:** Overview of Biopsies

Biopsy	Group	Sex	Age (years)	BV/TV (%)^a^	Time on pamidronate	Gene mutated	Comments	Reported in study
H1	Healthy control	F	19	24.62	X		^b^	Glorieux et al.^(^ ^26^ ^)^
H2	Healthy control	M	17	18.86	X		^b^	Glorieux et al.^(^ ^26^ ^)^
H3	Healthy control	M	20	29.69	X		^b^	Glorieux et al.^(^ ^26^ ^)^
H4	Healthy control	M	20	29.99	X		^b^	Glorieux et al.^(^ ^26^ ^)^
H5	Healthy control	F	20	34.32	X		^b^	Glorieux et al.^(^ ^26^ ^)^
H6	Healthy control	F	19	29.68	X		^b^	Glorieux et al.^(^ ^26^ ^)^
OI I 1	OI type I	M	17	22.90	6.4 years	COL1A1		Rauch et al.^(^ ^27^ ^)^
OI I 2	OI type I	F	20	20.27	5.3 years	COL1A1		Rauch et al.^(^ ^27^ ^)^
OI I 3	OI type I	F	19	36.34	X	COL1A1		Rauch et al.^(^ ^27^ ^)^
OI IV 1	OI type IV	M	20	16.38	5.0 years	COL1A2		Rauch et al.^(^ ^27^ ^)^
OI IV 2	OI type IV	F	20	10.20	5.3 years	COL1A1		Rauch et al.^(^ ^27^ ^)^
OI III 1	OI type III	F	19	7.07	4.7 years	COL1A2		Rauch et al.^(^ ^27^ ^)^

^a^ Reported bone volume/tissue volume (BV/TV) values are determined in the original study using histomorphometry.

^b^ No evidence of metabolic bone disease.

### Sample preparation

Since bone reveals an anisotropic elastic and post‐yield behavior at the ECM level, the biopsies had to be systematically oriented to perform the mechanical tests along the average osteonal axis of the cortical shell. Therefore, all biopsies were scanned using μCT (microCT 100, Scanco Medical AG, Bruttisellen, Switzerland) with a resolution of 10 μm (energy = 45 kVp, tube current = 200 μA, integration time: 300 ms). In each grayscale μCT image, a minimum of three Haversian channels were manually selected. These channels were segmented, and linear regression lines were fitted to the center of inertia of the segmented Haversian channels. Finally, out of the three regression lines, an average orientation was computed for each biopsy. A computer numerical controlled (CNC) milling program was used to fabricate a biopsy‐specific PMMA adapter with the correct orientation. Afterward, the biopsies were glued (UHU Endfest 300, Buhl, Germany) on the adapters to have their osteons aligned vertically. Finally, the oriented biopsies were milled (Polycut E Ultramiller, Leica, Wetzlar, Germany), lapped (Logitech PM5, Glasgow, UK) with a 1000 grit SiC powder, polished (Logitech PM5) with an ultra‐fine Al_2_O_3_ powder (grainsize 0.3 micron), and cleaned with DI water in an ultrasonic bath for 60 seconds to obtain a smooth surface normal to the longitudinal axis of the osteons.

### Tissue bone mineral density

Biopsies were rescanned using a hydroxyapatite calibrated μCT (microCT 100, Scanco Medical AG) with a resolution of 10 μm (energy = 45 kVp, tube current = 200 μA, integration time: 300 ms). Grayscale images were manually cropped, obtaining the cortical part of the biopsy using IPL (image processing language, Scanco Medical AG). The cropped images were first filtered (Gauss filter, σ = 0.8, support = 1) and then segmented with a previously defined threshold of 779.8 mgHA/cm^3^ to get the mask of the cortex. The threshold was defined by taking the average value of the automatically detected thresholds of the 12 biopsies using Otsu's method. The grayscale images were multiplied with the cortical mask receiving the masked cortical gray values. Finally, tissue bone mineral density (TMD) of the cortical bone was computed by the average of the masked cortical gray values. Filtering, segmentation, and matrix multiplication were done in Matlab R2018b (MathWorks, Natick, MA, USA).

### Micropillar compression

#### Micropillar fabrication

**Fig 2:**
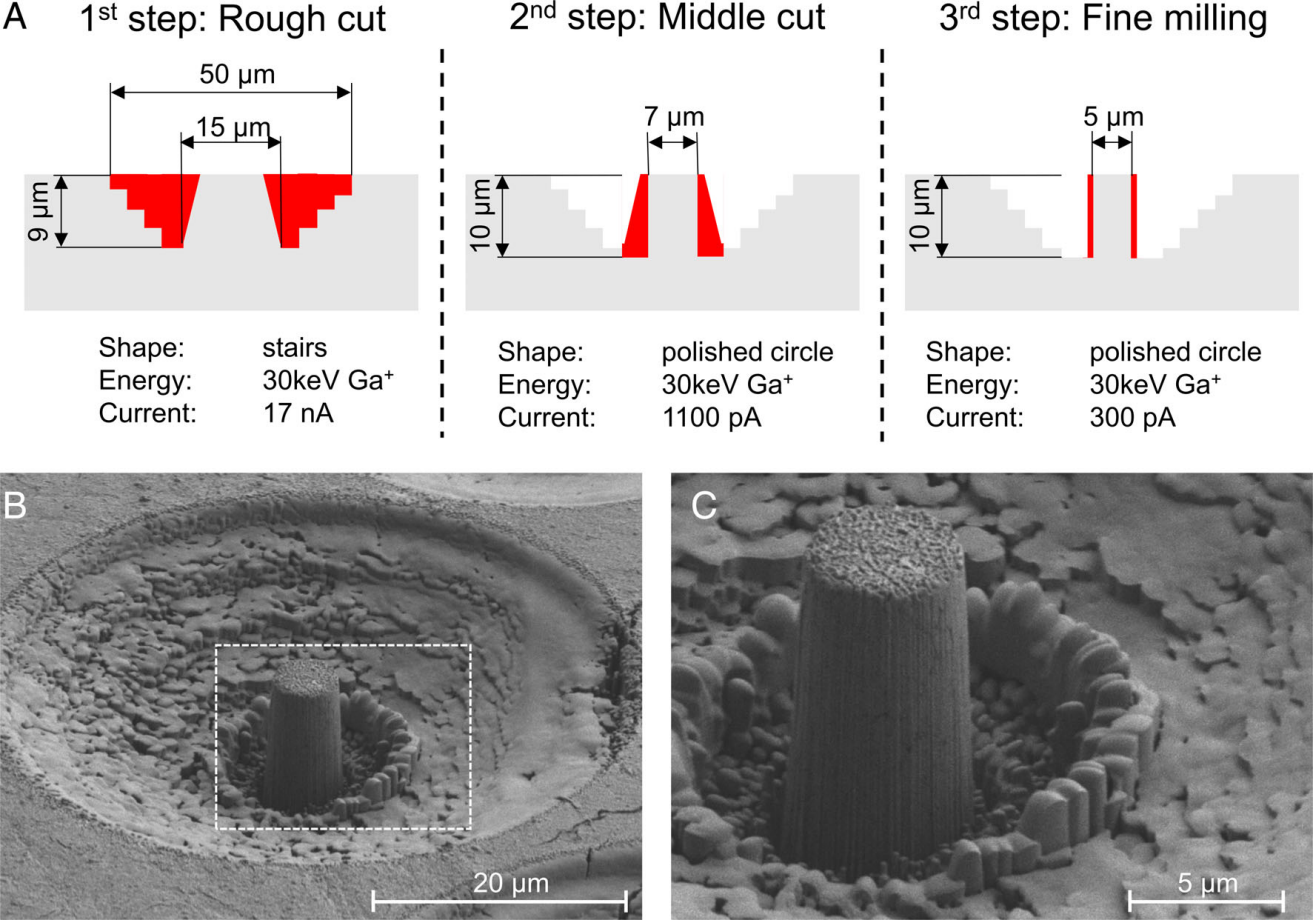
Pillar fabrication. (*A*) Three steps to produce micropillar with a geometry of approximately 5 μm in diameter and 10 μm in height. (*B*) Representative high‐resolution SEM image of a micropillar with trench and (*C*) with a higher magnification.

**Fig 3:**
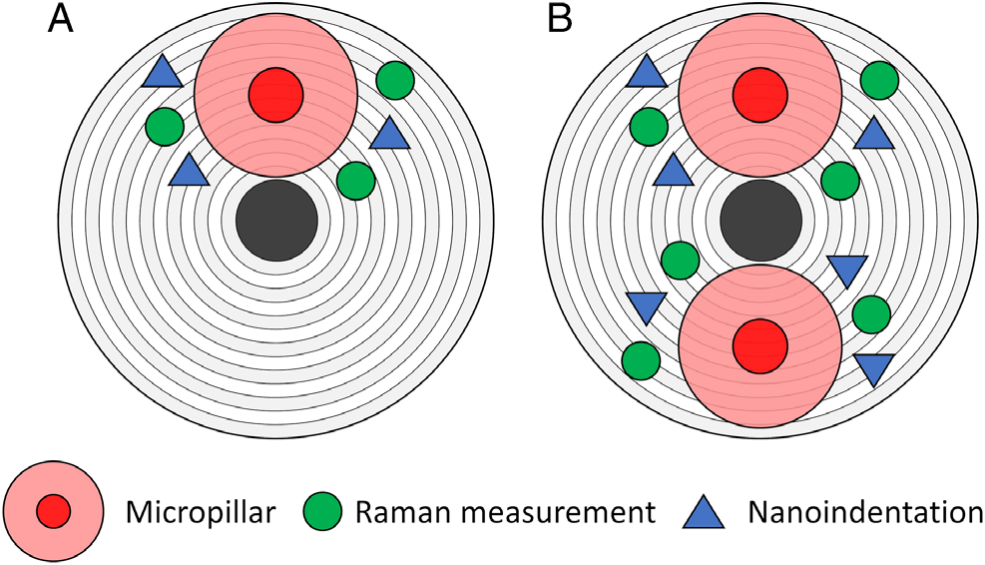
Scheme of the region of testing. (*A*) Around each micropillar, up to three Raman spectra and nanoindentation were performed (*B*) In some osteonal regions, multiple measurements were performed.

Micropillars were milled in vacuo using a dual beam instrument (Tescan Lyra, Brno, Czech Republic) consisting of focused ion beam (FIB) and scanning electron microscope (SEM). The embedded bone biopsies were sputtered with a thin gold film (11 nm) in a high vacuum sputter coater (Leica EM ACE600) to avoid drift due to electrical charging during the milling process. Pillars were manufactured according to a slightly modified protocol, developed by Schwiedrzik and colleagues.^(^ ^24^ ^)^ The final micropillars revealed a geometry of approximately 5 μm in diameter and 10 μm in height. The milling with Gallium ions (Ga^+^) was done in three steps (Fig. 2) and took about 1.5 hours per pillar. FIB milling can damage bone substrate due to Gallium implantation. Schwiedrzik and colleagues^(^ ^24^ ^)^ reported an implantation depth of Gallium ions into bone with 30 keV Ga^+^ up to 25 nm on the side of the micropillars (Monte Carlo simulation). Although ion damage occurs, the depth of 25 nm remains negligible. On the 12 transiliac crest bone biopsies, a total of 69 micropillars were milled in lamellar bone in osteonal regions (Fig. 3). Afterward, the exact geometry (diameter and height) of each micropillar was measured with a high‐resolution SEM (Hitachi [Tokyo, Japan] S‐4800, res. 9.9 nm).

#### Micropillar compression

Micropillars were compressed in vacuo inside an SEM (DSM962, ZEISS, Jena, Germany) using an in situ nanoindenter (Alemnis AG, Thun, Switzerland) equipped with a flat punch diamond tip (diameter = 10.8 μm, cone angle = 60°). The quasi‐static compression was controlled with a displacement rate of 5 nm/s that corresponds to a strain rate of approximately 5 10^−4^ s^−1^. During testing, displacement and reaction force were collected with a strain gauge‐based displacement sensor and load cell, respectively (recording frequency 10 Hz). A video of each experiment was recorded using the SEM to identify failure mechanisms. Three pillars failed in the manufacturing and testing phase and had to be excluded for the following reasons: There was a void inside the first pillar (probably an osteocyte inside); the second pillar was significantly weaker (probably no osteonal region); and the third pillar had to be aborted during testing because of a high drift of the loadcell. Thus, the total number of successfully tested micropillars is 66 (healthy/control *n* = 24; OI type I *n* = 16; OI type IV *n* = 11; and OI type III *n* = 15). The number of pillars tested per biopsy is given in Supplemental Table S1.

Compression tests reveal a toe region due to the progressive contact between the flat punch and the micropillar. This toe region is removed by searching for the highest stiffness in the loading part of the force‐displacement curves and shifting the displacement values so that the highest stiffness slope intersects with the origin. The machine compliance and the subsidence of the micropillar into the substrate were subtracted from the force‐displacement curve according to the improved Sneddon approach.^(^ ^28^ ^)^ The resulting force‐displacement curves were converted into engineering stress–strain curves and finally into true stress–logarithmic strain curves with the assumption that bone is incompressible. Equations used to derive the strain and stress are provided in the Supplemental Material.

Several parameters were extracted from the micropillar compression. The yield point was defined by the intersection point of the stress–strain curve and a 0.2% strain offset of the loading modulus. The ultimate point was determined as the point with the highest stress. For cross‐verification of micropillar compression with nanoindentation, the unloading modulus in the engineering regime was extracted similar to nanoindentation from the unloading part of the force‐displacement curve. A video of a representative OI type III micropillar compression test is provided in the Supplemental Material (time‐lapse video, 16 times faster).

### Raman spectroscopy

A confocal upright Raman spectroscope was used to measure the local degree of mineralization (Nova Spectra, ND‐MDT, Moscow, Russia). For this purpose, the biopsies were repolished (Logitech PM5) with an ultra‐fine Al_2_O_3_ powder (grain size 0.3 μm, polishing depth of approximately 1 to 2 μm) after micropillar compression to remove the gold film that was deposited for SEM imaging. After polishing, the trenches of the micropillars were still visible. Three Raman spectra were obtained in the periphery of the trenches (Fig. 3) and within the same osteon (exposure time: 60 seconds, wavelength = 633 nm, 50× objective [NA 0.75], spot size 1 μm in diameter). In total, 198 (66 × 3) Raman spectra were obtained. After background correction, the area integrations of the second phosphate peak (*v*_2_ *PO*_4_: 410–460 cm^−1^) and the *amideIII* peak (1215–1300 cm^−1^) were computed.^(^ ^29^ ^)^ The pillar‐specific mineral‐to‐matrix ratio was defined by the average of the three $\frac{v_{2}PO_{4}}{\text{amideIII}}$ measurements. The $\frac{v_{2}PO_{4}}{\text{amideIII}}$ ratio was chosen because of its independence of the local fibril orientation.^(^ ^30^ ^)^ In total, 66 averaged mineral‐to‐matrix ratios were collected (healthy/control *n* = 24; OI type I *n* = 16; OI type IV *n* = 11; and OI type III *n* = 15). The number of collected spectra per specimen is summarized in Supplemental Table S1.

### Nanoindentation

Three indentation measurements were performed around each micropillar (Fig. 3). The minimum distance between the indents and the trenches or other indents was 7 μm, preventing any deleterious interactions.^(^ ^18^ ^)^ Indentation was performed using a nanoindentation tester (Ultra Nano Hardness Tester, CSM Instruments, Peseux, Switzerland) equipped with a Berkovich diamond tip. A trapezoidal load control protocol was used following previous work.^(^ ^24^ ^)^ First, the tip was lowered with a loading rate of 100 mN/min to a depth of 1 μm. Second, the force was kept constant for 30 seconds. Last, the tip was unloaded with a rate of 400 mN/min. From a total of 196 indents (3 × 66), 14 nanoindentation measurements failed because of insufficient contact between tip and bone and had to be excluded from the study. An average value was computed with the available indents per pillar. Indentation modulus (*E* ^*^), hardness (*H*_*IT*_), dissipated work (*W*_*plas*_), and total work (*W*_*tot*_) were extracted according to standard methods.^(^ ^15^, ^18^ ^)^ Hardness‐indentation modulus ratio ($\frac{H_{\mathrm{IT}}}{E^{*}}$), which is a metric for yield strain, was also calculated.^(^ ^31^ ^)^ Relative dissipated work ($\frac{W_{\text{plas}}}{W_{\mathrm{tot}}}$) was defined as an indicator for strain hardening. In total, 66 averaged indentation data sets were computed (healthy/control *n* = 24; OI type I *n* = 16; OI type IV *n* = 11; and OI type III *n* = 15). The number of indentations per specimen is summarized in Supplemental Table S1.

### Statistics

All statistical analysis was performed in R (R version 3.6.1). The measurements were repeated within the biopsies and within the same osteonal region. Therefore, a mixed‐effect model was defined to detect differences within the groups (fixed effect) to reduce the inter (biopsy) and intra (osteonal region) variability. The biopsies and the nested osteons within the biopsies were taken as random effects. The model was fitted using the *lmer* function in the *lme4* package.

$$\text{variable}∼\text{Group}+\left(1|\text{Biopsy}\right)+\left(1|\text{Osteon}:\text{Biopsy}\right)$$

Significance of the group within the model was analyzed using a likelihood ratio test between a model with and without the fixed effect. Dunnett's post hoc test with the Bonferroni–Holm *p* value adjustment was used to detect significant changes between the OI types to the healthy control.

Because most of the analyzed variables show a similar trend, the mixed‐effect model was extended to a linear mixed‐effect model to analyze whether the variable (ultimate stress, yield strain, indentation modulus, and relative dissipated energy) is dependent on the degree of mineralization (covariance) on the groups (fixed effect) and on their interaction.

$$\text{variable∼}\frac{v_{2}PO_{4}}{\text{amideIII}}*\text{Group}+\left(1|\text{Biopsy}\right)+\left(1|\text{Osteon}:\text{Biopsy}\right)$$

Likelihood ratio tests between the linear mixed‐effect model with and without the fixed effect were used to identify the influence of the group. Finally, linear regression models were fitted between different physical measurements averaged on biopsies or on sites. Adjusted *R* ^2^ were computed, and *F*‐statistics was used to check the quality of the fit. Confidence and prediction bands were plotted. The level of significance was set to 95% (*p* < 0.05).

## Results

On 12 transiliac crest bone biopsies (healthy/control *n* = 6; OI type I *n* = 3; OI type IV *n* = 2; and OI type III *n* = 1) four different measurements techniques (μCT, Raman spectroscopy, micropillar compression test, and nanoindentation) were analyzed, and the results are summarized in Table 2.

**Table 2:** Mean and Standard Deviation of the Different Observed Variables of the Four Groups

Methods	Variables	Likelihood ratio test	Healthy/control	OI type I	OI type IV	OI type III
μCT	No. of observations		*n* = 6	*n* = 3	*n* = 2	*n* = 1
	$\mathrm{TMD}\;\left[\frac{\text{mgHA}}{cm^{3}}\right]$ ^a^	N/A	1012.7 ± 41.6	1097.6 ± 17.0	1143.9 ± 42.2	1141.1
Raman	No. of observations		*n* = 24	*n* = 16	*n* = 11	*n* = 15
	$\frac{v_{2}PO_{4}}{\text{amideIII}}\left[-\right]$	*p* = .0014	0.351 ± 0.119	0.543 ± 0.093 (*p* = .016)	0.690 ± 0.132 (*p* = .00016)	0.615 ± 0.133 (*p* = .018)
Micropillar compression	No. of observations		*n* = 24	*n* = 16	*n* = 11	*n* = 15
	*σ*^*ult*^ [*GPa*]	*p* = .033	0.590 ± 0.128	0.716 ± 0.076 (NS)	0.789 ± 0.071 (*p* = .0461)	0.770 ± 0.121 (NS)
	*ε*^*ult*^ [−]	*p* = .314	0.090 ± 0.035	0.097 ± 0.046 (NS)	0.115 ± 0.042 (NS)	0.142 ± 0.047 (NS)
	*σ*^*yield*^ [*GPa*]	*p* = .341	0.350 ± 0.097	0.419 ± 0.075 (NS)	0.446 ± 0.078 (NS)	0.397 ± 0.086 (NS)
	*ε*^*yield*^[−]	*p* = .263	0.026 ± 0.003	0.027 ± 0.002 (NS)	0.028 ± 0.003 (NS)	0.024 ± 0.002 (NS)
Nano indentation	No. of observations		*n* = 24	*n* = 16	*n* = 11	*n* = 15
	E^*^ [*GPA*]	*p* = .062	19.8 ± 2.22	23.8 ± 3.04 (NS)	22.9 ± 1.87 (NS)	22.0 ± 1.98 (NS)
	*H*_*IT*_ [*MPa*]	*p* = .0033	699 ± 77.5	825 ± 134 (*p* = .022)	913 ± 98.7 (*p* = .00102)	816 ± 165 (0.031)
	$\frac{H_{\mathrm{IT}}}{E^{*}}\;\left[-\right]$	*p* = .034	0.036 ± 0.003	0.035 ± 0.002 (NS)	0.040 ± 0.002 (*p* = .027)	0.037 ± .004 (NS)
	*W*_*tot*_ [*pJ*]	*p* = .0091	7067 ± 979	8224 ± 1111 (*p* = .037)	8803 ± 663 (*p* = .0092)	8152 ± 1420 (*p* = .045)
	*W*_*plas*_ [*pJ*]	*p* = .0292	5515 ± 778	6411 ± 817 (NS)	6635 ± 445 (NS)	6201 ± 875 (NS)
	$\frac{W_{\text{plas}}}{W_{\mathrm{tot}}}\;\left[-\right]$	*p* = .0034	0.78 ± 0.014	0.78 ± 0.01 (NS)	0.754 ± 0.015 (*p* = .0019)	0.765 ± 0.028 (*p* = .447)

In parentheses are the *p* values of the Dunnett's post hoc test. In the third column are the *p* values of the likelihood ratio test of the mixed‐effect model with and without the group as a fixed effect.

^a^ No statistics available because of small sample size.

### Degree of mineralization

The TMD value of μCT is increased for all OI groups compared with healthy control. Because of the low sample sizes, no statistical analysis could be performed. The mineral‐to‐matrix ratio of Raman spectroscopy is significantly different among the groups (*p* = .014). The Dunnett's test revealed that all three OI types were more highly mineralized compared with healthy control (OI type I: *p* = .016; OI type IV: *p* = .0016; and OI type III: *p* = .018) (Fig. 5*A*).

### Mechanical properties of micropillar compression

**Fig 4:**
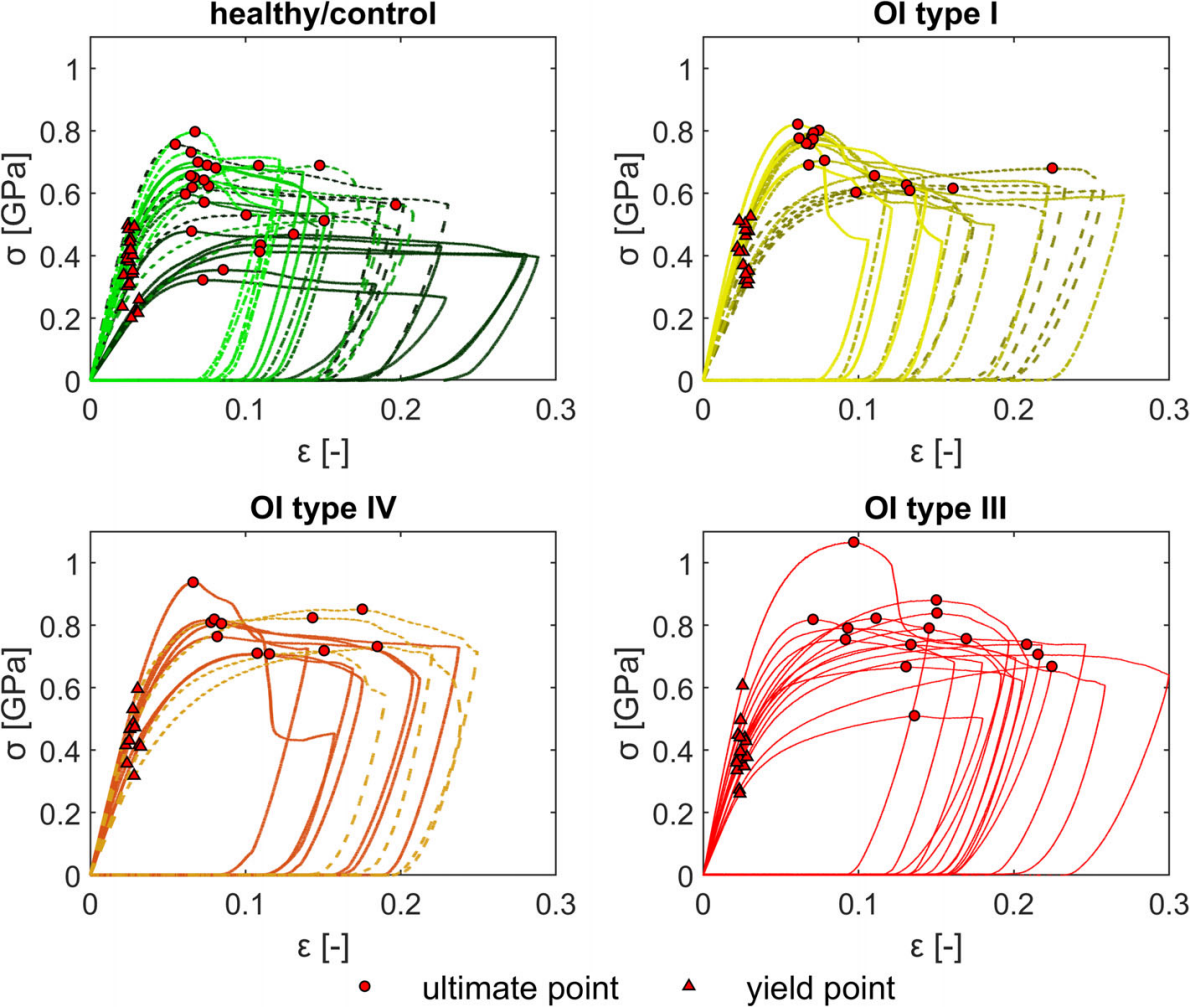
Stress–strain curves of micropillar compression test with the yield point (red triangle) and point of ultimate strength (red dot). The different biopsies are marked with different line types and colors.

Qualitatively, the stress–strain curves of the micropillar compression test reveal no obvious difference in the mechanical behavior of bone ECM between healthy control and the different types of OI (Fig. 4). Nevertheless, there is high heterogeneity, especially of the post‐yield properties between the biopsies. In some cases, stress decreases after a sharp maximum stress at a strain typically lower than 10%, which is designated by strain softening and, in other cases, the stress increases continuously after yielding and reaches a maximum at strains higher than 10%, which is called strain hardening.

**Fig 5:**
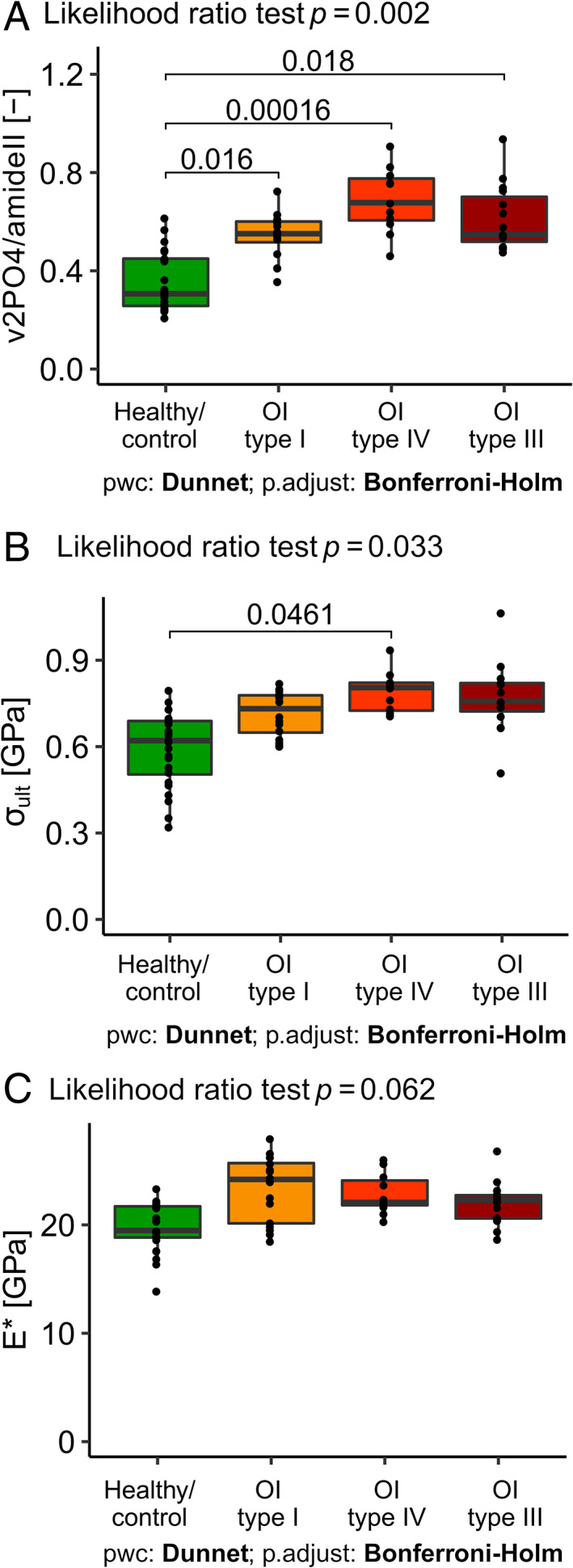
Comparison between groups of (*A*) mineral‐to‐matrix ratio (*v*_2_ *PO*_4_/*amideIII*), (*B*) ultimate stress (σ_ult_), and (*C*) indentation modulus (E^*^).

The likelihood ratio test revealed that the ultimate stress is significantly different between the groups (Table 2). Although all three OI groups are higher in the mean, the post hoc test showed that only the ultimate stress of OI type IV is significantly (*p* = .0461) higher compared with the healthy control group (Fig. 5*B*). The statistical tests did not detect any significant difference between ultimate strain, yield stress, and yield strain. However, the ultimate strain and yield stress tend to be higher for all OI types. This trend is not visible for yield strain.

### Mechanical properties of nanoindentation

The indentation modulus tends to be higher for all OI types compared with healthy control, although none of that significantly (Fig. 5*C*). However, hardness is significantly superior for all OI groups compared with healthy control (Supplemental Fig. S2*A*). Only the $\frac{H_{\mathrm{IT}}}{E^{*}}$ ratio of OI type IV is significantly higher compared with the healthy control (*p* = .027) (Supplemental Fig. S2*B*). The total energy is superior for all OI groups compared with healthy control (OI type I: *p* = .037; OI type IV: *p* = .0092; and OI type III: *p* = .045). As well for the dissipated energy, all OI groups tend to be higher compared with healthy control, although not significantly. The mean relative dissipated energy is equal for OI type I compared with healthy control; however, only OI type IV (*p* = .0014) and OI type III (*p* = .0447) are significantly smaller compared with healthy control (Supplemental Fig. S2*C*).

### Correlation between methods

**Fig 6:**
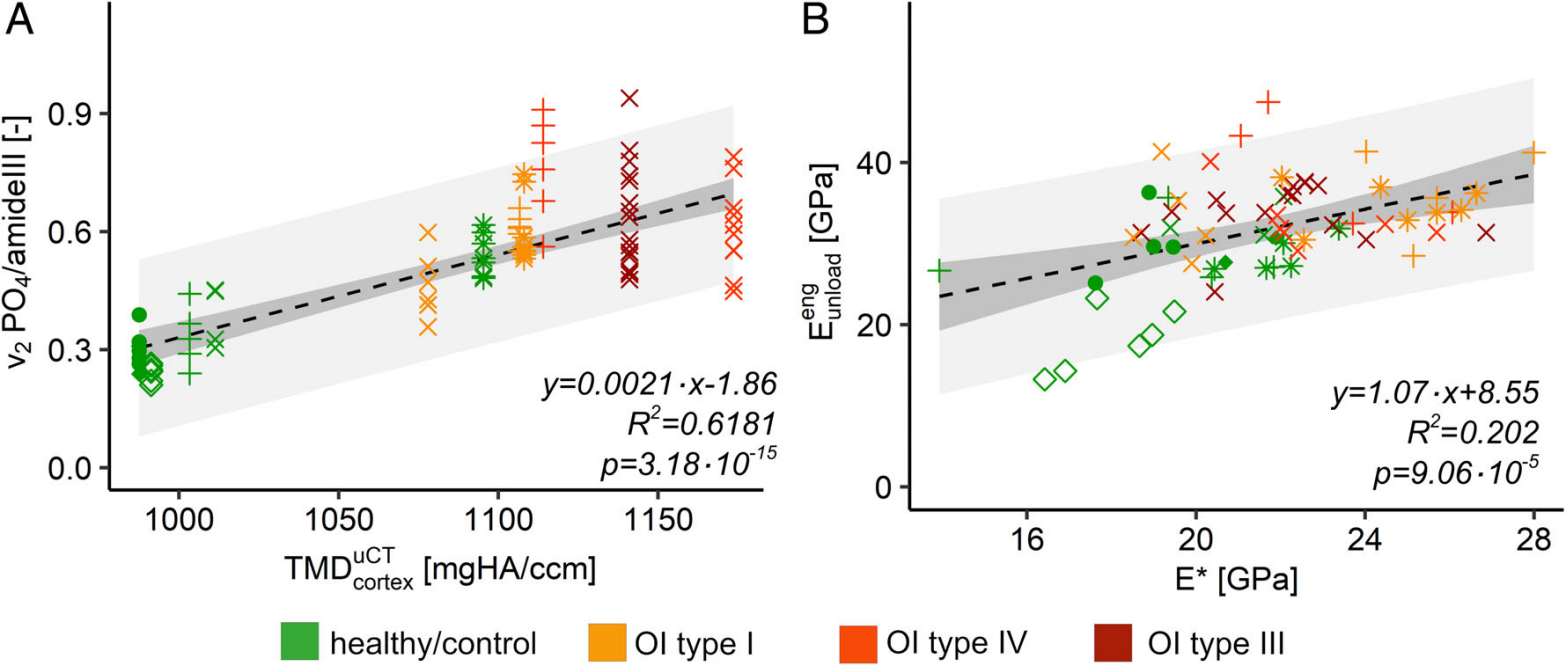
Comparisons between methods. (*A*) Mineral‐to‐matrix ratio (*v*_2_ *PO*_4_/*amideIII*) versus tissue mineral density (TMD) of the cortical shell. (*B*) Unloading modulus of micropillar compression test versus indentation modulus of nanoindentation (E^*^). Different point shape = different biopsy; dashed black line = regression curve of the linear model; dark gray = confidential interval (95%); light gray = prediction interval (95%).

The degree of mineralization was measured with two different methods. Mineral‐to‐matrix ratio measured by Raman spectroscopy and TMD measured by μCT correlate linearly (Fig. 6*A*, linear model, *F*‐test: *p* < .0001). The mechanical properties were captured with two techniques. Unloading modulus of micropillar compression test and indentation modulus of nanoindentation (Fig. 6*B*, linear model, *F*‐test: *p* < .0001) correlate linearly.

### Degree of mineralization versus mechanical properties

**Fig 7:**
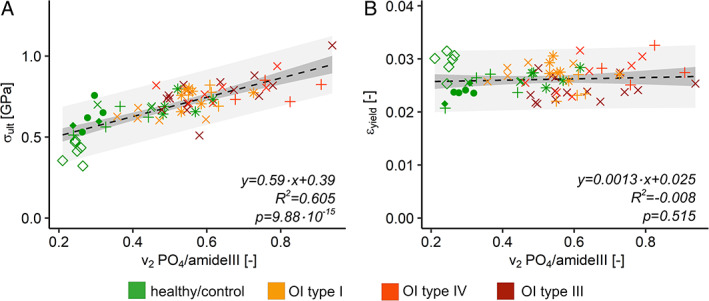
Mechanical parameters of micropillar compression versus degree of mineralization. (*A*) Ultimate stress of micropillar compression (σ_ult_) in relation to mineral‐to‐matrix ratio ($\frac{v_{2}PO_{4}}{\text{amideIII}})$. (*B*) Yield strain of micropillar compression (ε_yield_) in relation to mineral‐to‐matrix ratio ($\frac{v_{2}PO_{4}}{\text{amideIII}})$. Different point shape = different biopsy; dashed black line = regression curve of the linear model; dark gray = confidential interval (95%); light gray = prediction interval (95%).

The likelihood ratio test between the linear mixed‐effect model with and without the group revealed that the OI groups do not have a significant effect on the model. Ultimate stress is only dependent on the degree of mineralization. Ultimate stresses from micropillar compression increase linearly with mineral‐to‐matrix ratio (Fig. 7*A*, linear model, *F*‐test: *p* < .0001). In contrast, yield strains are neither dependent on the degree of mineralization nor on the groups, which is also visible for the linear model in Fig. 7*B* (linear model *F*‐test: *p* > .05).

**Fig 8:**
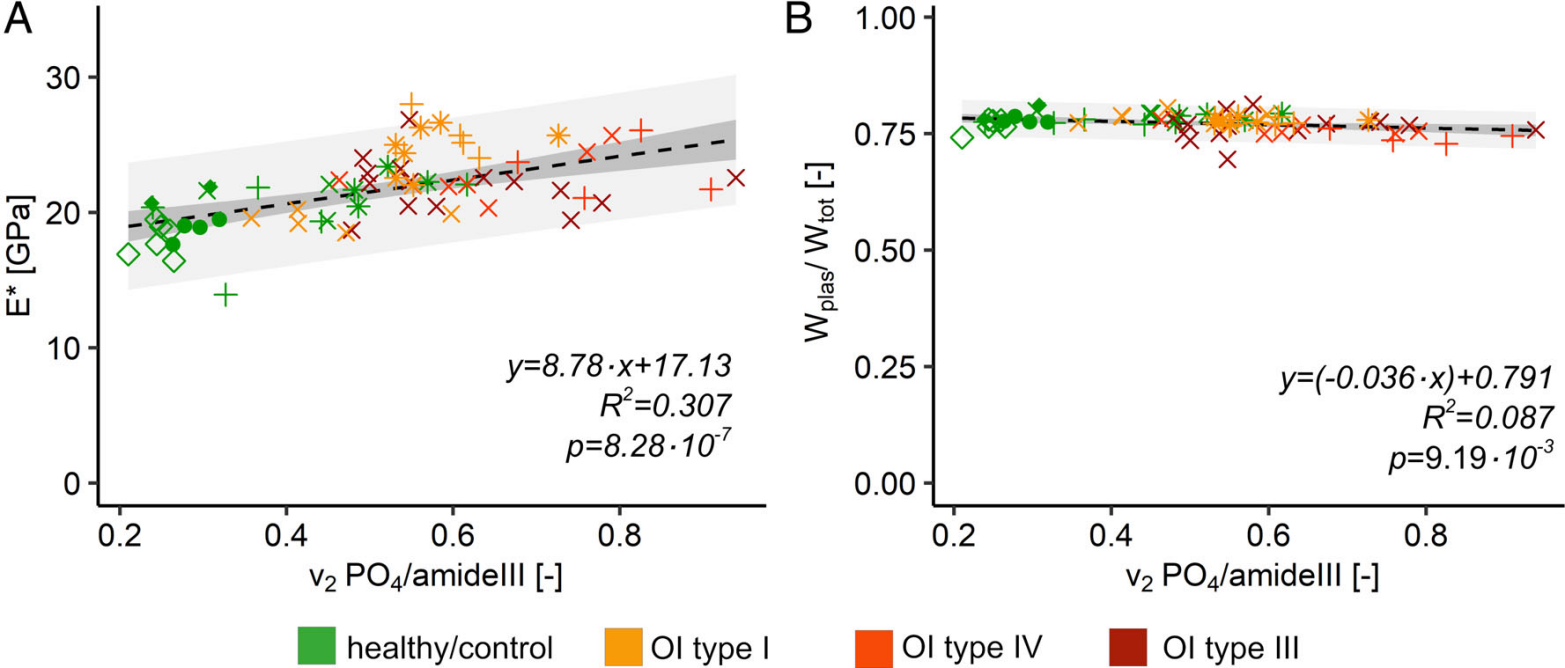
Mechanical parameters of nanoindentation versus the degree of mineralization. (*A*) Indentation modulus of nanoindentation (E^*^) in relation to mineral‐to‐matrix ration ($\frac{v_{2}PO_{4}}{\text{amideIII}})$. (*B*) Relative dissipated work of nanoindentation ($\frac{W_{\text{plas}}}{W_{\mathrm{tot}}}$) in relation to mineral‐to‐matrix ratio ($\frac{v_{2}PO_{4}}{\text{amideIII}}).$ Different point shape = different biopsy; dashed black line = regression curve of the linear model; dark gray = confidential interval (95%); light gray = prediction interval (95%).

The likelihood ratio test disclosed that the group does not have a significant influence on the linear mixed‐effect model for the indentation modulus. The linear model without the random effect between indentation modulus and the degree of mineralization is highly significant and the modulus increases with higher degree of mineralization (Fig. 8*A*, linear model *p* < .0001). For the relative plastic indentation work, the likelihood ratio test detected a significant difference between the model with and without the groups (*p* = .007). Further analyses showed that the interaction between OI type IV and the degree of mineralization is significantly lower compared with the other interactions (*p* = .02). Interestingly, the relative plastic indentation work decreases slightly but significantly with increasing degree of mineralization (Fig. 8*B*, linear model *p* = .009).

## Discussion

Bone mechanical behavior results from deformation and fracture of bone's multiscale structure. The present study aimed to compare the compressive elastic and post‐yield properties of bone ECM from young adults with three different OI types to a healthy control group. The relationships between the measured mechanical properties and the degree of mineralization were then investigated. Our working hypotheses were that (i) the compressive mechanical properties at the ECM level are inferior in OI bone than in healthy bone, and (ii) elastic modulus, yield, and ultimate strength but also brittleness of bone ECM increase with the degree of mineralization.

### Degree of mineralization

Degree of mineralization was measured with two different techniques. Global assessment of the mineralization was performed by a hydroxyapatite calibrated μCT, which directly measures the mass of bone mineral. The local assessment of the degree of mineralization was done by Raman spectroscopy, where the relative amount of mineral over collagen in a given volume is measured, also called mineral‐to‐matrix ratio. Roschger and colleagues showed that the mineral‐to‐matrix ratio correlates linearly with calcium weight fraction (*R* ^2^ = 0.75).^(^ ^29^ ^)^ OI bone shows a significantly higher mineralization level than healthy bone (Table 2), as measured by Raman spectroscopy, which confirms the literature.^(^ ^6^, ^7^, ^8^, ^9^ ^)^ The TMD of cortical shell measured with μCT for healthy control and OI bone in the present study are in agreement with values reported in the literature (control: 988^(^ ^6^ ^)^ and 1032^(^ ^7^ ^)^ mgHA/cm^3^; OI: 1103^(^ ^6^ ^)^ and 1131^(^ ^7^ ^)^ mgHA/cm^3^). The relationship between the global cortical TMD values and the local mineral‐to‐matrix ratio revealed a highly significant correlation (*p* < .0001). Raman spectroscopy measures the mineral‐to‐matrix ratio on a spot size of approximately 1 micron in diameter, while μCT is limited to quantify an average mineralization of the cortical shell of a biopsy at the tissue level because of its limited resolution, noise, and partial volume effect.

The biopsies were acquired from OI patients who had previously received bisphosphonate treatment (pamidronate), which is standard clinical practice in the treatment of growing children with OI.^(^ ^32^ ^)^ One individual with OI type I included in our study (OI I 3) did not receive any treatment. This untreated OI biopsy shows a similar mineralization (TMD_OI1 3_ = 1106.7 mgHA/cm^3^) compared with the other two OI type I biopsies (TMD_OI1 1_ = 1077.9 mgHA/cm^3^ and TMD_OI1 2_ = 1108.0 mgHA/cm^3^) and the potential influence of bisphosphonate treatment on tissue mineralization remains therefore unclear. Weber and colleagues reported that pamidronate does not alter the mineralization and the mechanical properties of pediatric OI bone during a treatment period of approximately 2.5 years on pediatric patients.^(^ ^8^ ^)^ Unfortunately, the statistical quantification of the finding of Weber and colleagues was not possible in the present study because of the small sample size.

### Mechanical properties in healthy controls

In healthy control bone, our measurements of compressive behavior at the ECM level give an indentation modulus of 19.8 ± 2.2 GPa (*n* = 24), an ultimate stress of 590 ± 128 MPa (*n* = 24), an ultimate strain of 9 ± 3.5% (*n* = 24), a yield stress of 350 ± 97 GPa (*n* = 24), and a yield strain of 2.6 ± 0.3% (*n* = 24). At the ECM scale, bone shows a ductile post‐yield behavior (Fig. 4). Although measured in trabecular bone, Tertuliano and colleagues reported a similar yield strength of 327 ± 20 MPa using micropillar compression (d = 3 μm) at 5.1% ± 0.9% strain in human, dry, ordered lamellar bone.^(^ ^33^ ^)^ Macroscopic compression testing of dry human cortical bone was reported by Evans^(^ ^34^ ^)^ to exhibit an elastic and a quasi‐brittle post‐yield behavior, with a Young's modulus of 17.92 ± 1.5 GPA (*n* = 5)^(^ ^35^ ^)^ and an ultimate stress of 177.1 ± 32.7 MPa (*n* = 38)^(^ ^35^ ^)^ in the longitudinal direction. Comparing the two length scales, Young's modulus is approximately 10% lower at the macroscopic scale compared with the isotropic indentation modulus, which can be explained by the lacunar‐canalicular and Haversian porosity.^(^ ^36^ ^)^ However, the macroscopic ultimate stress is 3.3 times smaller than the ECM levels. Similar findings were found in ovine bone^(^ ^24^ ^)^ and can be explained by the stress concentrations produced by resorption spaces, lacunar and vascular porosity, defects, microcracks, and interfaces present in mm‐sized samples and turn bone into a quasi‐brittle material at the macroscopic level.

### Mechanical properties in OI

Interestingly, in the OI ECM level compression tests, qualitative examination of the stress–strain curves (Fig. 4) reveals that OI bone does not exhibit lower strength and does not behave in a more brittle manner than the ECM of healthy bone. Furthermore, the micropillars of healthy and OI bone show a heterogenous, post‐yield behavior in terms of strain softening or hardening, which is also represented in the relatively high standard deviations within the groups. Schwiedrzik and colleagues reported an anisotropic post‐yield behavior for axial (strain softening) and transverse (strain hardening) loading with respect to the collagen fibers orientation of the ovine tibia.^(^ ^24^ ^)^ Kochetkova and colleagues reported that the change of strain hardening to softening occurs around a 50° angle of the collagen with respect to the loading axis and the failure mechanism is highly dependent on this angle.^(^ ^37^ ^)^ The heterogeneity of the micropillar compression curves in the present study may be explained first by the less regular osteonal organization of iliac crest bone compared with ovine tibia (long bone) and second by the twisted plywood organization of the lamellar architecture within a human osteon.

Notably, micropillar compression revealed higher ultimate stresses at the ECM level of each OI group compared with healthy controls. Furthermore, the ultimate stresses at the ECM level increased with OI severity. The mean ultimate strain increased with OI severity; however, the relative distributions of ultimate strain within the groups are relatively high. This broad distribution of the ultimate strain is explained by the anisotropic post‐yield behavior (strain softening and hardening).^(^ ^24^ ^)^ Furthermore, mean yield stress is not significantly different within the groups. Yield strain was equal among the groups.

Nanoindentation was performed for comparison to previous studies. The indentation modulus of OI bone tends to be higher compared with healthy control, whereas hardness of OI bone is significantly higher than healthy control. Both, indentation modulus, and hardness are in the same range as reported in literature for transiliac crest biopsies from OI individuals treated with pamidronate.^(^ ^8^ ^)^ The results confirm the findings of Fan and colleagues that the indentation modulus and hardness of OI type III and OI type IV are not significantly different.^(^ ^20^ ^)^ Albert and colleagues reported a significantly higher indentation modulus for OI type I compared with OI type III;^(^ ^19^ ^)^ in the present study, the mean indentation modulus of OI type I (+6.33%) is higher than OI type III, but the difference is not significant. The hardness‐indentation modulus ratio $\left(\frac{H_{\mathrm{IT}}}{E^{*}}\right)$ is an indicator of the yield behavior. Only OI type IV is significantly higher than the healthy control. Furthermore, the plastic and total work is higher for OI bone compared with healthy control. A decreasing relative dissipated energy $\left(\frac{W_{\text{plas}}}{W_{\mathrm{tot}}}\;\right)$ is an indicator for increasing strain hardening of the bone with mineralization. This relative dissipated energy differs significantly within the four groups and the mean value is decreasing with OI severity compared with healthy control. Statistically, the relative dissipated energy from indentation follows the trend of ultimate stress from micropillar compression (Table 2), which reflects the connection between the extent of strain hardening and ultimate stress in a stress–strain diagram. In contrast to a widespread belief, this finding suggests that an increase in bone mineralization within the range of normal and OI bone is not associated with an increased brittleness in compression.

### Relationship between degree of mineralization and mechanical properties

Most of the analyzed parameters (mineralization and mechanical) correlate positively or negatively with OI severity. Linear models were used to fit the mechanical properties with the degree of mineralization. Ultimate stress is highly dependent on the degree of mineralization (*p* = 9.88 × 10^−15^). However, the yield strain is independent of the degree of mineralization (*p* = .515). Indentation modulus is increasing with a positive slope (*p* = 3.79 × 10^−9^) and the relative dissipated energy, a metric for strain hardening, is slightly decreasing with higher degree of mineralization (*p* = 6.93 × 10^−4^). On the other hand, the models deliver a relatively poor *R* ^2^ and a large prediction band. Because iliac crest bone is heterogeneous at the ECM level and differs within the lamellae, it is not possible to collect properties of the exact same spot. Furthermore, mineral‐to‐matrix ratio and indentation values were average values of up to three collected measurements. Although the models reveal low *R* ^2^ values, they predict the trends with a highly significant *p* value.

The degree of mineralization dominates mechanical properties of the bone ECM with respect to the control and OI groups. The increase of elastic modulus with mineralization is predicted by about any computational model of bone ECM (eg, Alizadeh and colleagues^(^ ^38^ ^)^), when the organic components maintain the same material properties, but this is presumably not the case in OI. However, in compression, the mechanical role of type I collagen is merely a soft filler between stiff mineral crystals and may not be significantly altered by molecular defects. On the other hand, OI bone ECM seems to achieve higher degrees of mineralization, which may precisely be due to the molecular defects rather than bisphosphonate treatment. Accordingly, OI bone has stronger compressive mechanical properties, which is in complete contradiction with the high prevalence of fractures in OI patients. However, many fractures may be triggered by tensile stresses, and we know that post‐yield behavior of bone ECM is driven by different mechanisms in tension and compression.^(^ ^39^ ^)^ The collagen defects associated with OI may theoretically have a more profound influence on tensile than on compressive properties.

Nevertheless, the mesoscale organization of bone tissue may also contribute to bone fragility in OI at the macroscopic scale. The average bone volume fraction (BV/TV) for the used biopsies (reported in two previous studies;^(^ ^26^, ^27^ ^)^ Table 1) is decreasing with OI severity (healthy control = 27.9% ± 5.4%; OI type I = 26.5% ± 8.6%; OI type IV = 13.3% ± 4.4%; and OI type III = 7.1%). As shown by numerous studies, porosity plays a major role in toughness of aging bone and operates above the considered ECM level (eg, Zimmermann and colleagues^(^ ^40^ ^)^). As suggested by the much higher ultimate strains of the ECM level compared with the macroscopic level, the apparent mechanical benefit of a higher mineralization at the ECM level could be easily reversed by deleterious pore distributions or thin cortical walls, which would reconcile the contradiction formulated above.

### Limitations of the study

The mechanical testing has some methodological limitations. Although we are reporting values in the longitudinal direction, the biopsies contained only a few vascular canals to determine the osteon orientation, which represents a first limitation. It has been shown in several studies that the mechanical properties are dependent on the orientation of the collagen fibers.^(^ ^18^, ^24^, ^25^, ^37^, ^39^ ^)^ Unfortunately, due to pillar fabrication using FIB (Gallium) and the destructive nature of the microcompression tests, it was not anymore possible to measure the collagen orientation in the specimen at the position of the micropillar using a technique such as polarized light. However, the careful orientation of our samples along the osteonal canals and the similar appearance/variability of the post‐yield behavior among the groups exclude a systematic bias of the mechanical properties due to collagen orientation.

A further limitation is the in vacuo and dry testing conditions, which does not match physiological conditions. Schwiedrzik and colleagues showed that ovine bone in the longitudinal direction for the hydrated state has a 2.88 times lower yield stress and a 4.16 times lower ultimate stress compared with in vacuo testing conditions.^(^ ^25^ ^)^ The wet indentation modulus, hardness, elastic, and total work in axial direction is 1.21, 1.68, 1.70, and 1.66 times smaller, respectively, compared with dry conditions.^(^ ^24^ ^)^ Considering swelling of the organic phase with the addition of water in a nested shear lag model,^(^ ^38^ ^)^ we expect that the linear relationship between mineral content and longitudinal elastic properties observed in dry bone exhibits a reduced slope and intercept but remains significant for wet bone (Supplemental Material). Nevertheless, despite the difficulty of obtaining fresh OI tissue, experimental verification on wet OI bone will become necessary to resolve this issue. Lastly, this study reports the properties of fixed human bone. In an internal, yet unpublished study, two ovine bone specimens were tested in fixed and unfixed conditions, and the results showed that the fixation does not alter the mineral‐to‐matrix ratio (Raman spectroscopy) and the mechanical properties measured in dry condition (micropillar and nanoindentation). Additionally, Florez and colleagues also showed that the PMMA infiltration during the fixation process on bone in mice does not change the modulus, but hardness is significantly increased, since nanopores are filled with PMMA.^(^ ^41^ ^)^

Apart from the above‐mentioned methodological limitations, one of the major limitations is the low number of biopsies and its statistical power. Although transiliac biopsies are difficult to obtain because of the invasive nature of the procedure, they have the advantage of being standardized in terms of size and anatomical location compared with bone material removed during a surgical intervention. Furthermore, the relatively low number of tested micropillars is due to the considerable effort to manufacture them. Another limitation is the confounding bisphosphonate (pamidronate) treatment, which is the standard of care in these patients. Only a single OI type I biopsy was free of treatment. Thus, the influence of bisphosphonate treatment on OI bone ECM could not be analyzed. Lastly, the present study did not include tensile loading, but micropillar testing brings additional information to nanoindentation, especially regarding post‐yield properties.

## Conclusion

In conclusion, the ECM‐level compressive mechanical properties of OI bone showed a trend to higher modulus, ultimate stress, and post‐yield behavior than healthy bone. We found strong relationships between the degree of mineralization and both the ECM‐level elastic modulus and the ultimate strength of bone in compression. The broad range of degree of mineralization observed in this study allows us to extend the above findings to strain hardening. Our results do not demonstrate brittleness of OI bones in ECM‐level compressive material properties. Indeed, brittleness may stem from poor bone organization at larger length scales or deficient post‐yield behavior in tension at the ECM level, which may be a dominant fracture mode in OI patients.

## Disclosures

MI and DC acknowledge the Swiss National Science Foundation (SNF grant #165510) for the financial support. MI also acknowledges Mereo BioPharma for financial support. CP and JS acknowledge funding through SNF Ambizione grant no. 174192. TK acknowledges funding through SFA PHRT IDoc grant no. 2017–304. PZ, BMW, and FR have received institutional research support and materials and are consultants for Mereo BioPharma.

### Peer Review

The peer review history for this article is available at https://publons.com/publon/10.1002/jbmr.4286.

## Supporting information

**Appendix S1.** Supplemental Information

## Data Availability

Data available on request from the authors.
