# Supplementary material for: Compressive Strength of Iliac Bone ECM Is Not Reduced in Osteogenesis Imperfecta and Increases With Mineralization
Source: J Bone Miner Res. 2021 Apr 4;36(7):1364–75. doi: 10.1002/jbmr.4286 (PMC8359849; doi:10.1002/jbmr.4286)
Supplement: Supplementary file 1 — Appendix S1. Supplemental Information [file JBMR-36-1364-s001.zip › JBMR_4286_Supplementary_material_R2_v1.docx]

Compressive strength of iliac bone ECM is not reduced in Osteogenesis Imperfecta and increases with mineralization

Michael Indermaur^a^, Daniele Casari^b^, Tatiana Kochetkova^b^, Cinzia Peruzzi^b^, Elizabeth Zimmermann^c,d^, Frank Rauch^c,d^, Bettina Willie^c,d^, Johann Michler^b^, Jakob Schwiedrzik^b^, Philippe Zysset^a^

1. ARTORG Center for Biomedical Engineering Research, University of Bern, Bern, Switzerland
2. Swiss Federal Laboratories for Material Science and Technology, Empa, Thun, Switzerland
3. Shriners Hospital for Children, Montreal, Canada
4. McGill University, Montreal, Canada

**Supplementary materials**

# Micropillar compression equations

Following equations were used to derive the strain and stress curves. First the machine compliance ($C_{mach compliance}$) was subtracted from the total compliance ($C_{total}$) receiving a machine compliance corrected compliance ($C_{mach cor}$).

| $C_{mach cor}=C_{total}-C_{mach compliance}$ | Eq. 1 |
| --- | --- |
| $C_{mach cor}=C_{total}-0.0042\frac{\mu m}{mN}$ | Eq. 2 |

So, the machine compliance corrected displacement ($U_{mash cor}$) can be determined:

| $U_{mach cor}=U-0.0042\frac{\mu m}{mN} F$ | Eq. 3 |
| --- | --- |

The $C_{mach cor}$ is the sum of the compliance of the pillar ($C_{pillar}$) and the compliance of the pillar subsidence into the substrate ($C_{improved sneddon}$), defined by the modified Sneddon approach of Zhang et al.^(1)^. $C_{improved sneddon}$ contains the Poisson ratio $\nu$, the Young’s modulus $E$ and the $a_{c}$:

| $C_{mach cor}=C_{pillar}+C_{improved sneddon}$ | Eq. 4 |
| --- | --- |
| $\frac{U_{mach cor}}{F}=\frac{U_{pillar}}{F}+\frac{1-\nu^{2}}{2Ea_{c}}$ | Eq. 5 |
| With: $a_{c}=\eta\left( r+r_{c} \right)$, $\eta$=1.42 according to Zhang et al.^(1)^  $r$ radius of the pillar  $r_{c}$ was assumed to be 0.3 µm |  |

Thus, Young’s modulus ($E$) is defined by the following formula:

| $E=\frac{-0.5 F \left( \upsilon^{2}-1 \right)}{a_{c}\left( U_{mach cor}-U_{pillar} \right)}$ | Eq. 6 |
| --- | --- |

According to Hooke’s law, the displacement of the pillar ($U_{pillar}$) is defined by:

| $U_{pillar}=\frac{FL_{pillar}}{EA_{pillar}}$ | Eq. 7 |
| --- | --- |

Using Eq. 6 and Eq. 7, $U_{pillar}$can be described by Eq. 8

| $U_{pillar}=\frac{-2L_{pillar} a_{c} U_{mach cor}}{A_{pillar}\upsilon^{2}-A_{pillar}-2 L_{pillar} a_{c}}$ | Eq. 8 |
| --- | --- |

Thus, the engineering strain is defined by $U_{pillar}$ over the initial pillar length ($L_{pillar}$)

| $\varepsilon_{eng}=\frac{U_{pillar}}{L_{pillar}}=\frac{-2 a_{c} U_{mach comp}}{A_{pillar} \nu^{2}-A_{pillar}-2 L_{pillar} a_{c}}$ | Eq. 9 |
| --- | --- |

And the engineering stress is defined as followed:

| $\sigma_{eng}=\frac{F}{A_{pillar}}$ | Eq. 10 |
| --- | --- |

Lastly, the engineering stress and strain were converted into true stress $\sigma$ and logarithmic strain$\varepsilon$ with the approximation that bone is incompressible:

| $\sigma=\sigma_{eng}(1+\varepsilon_{eng})$ | Eq. 11 |
| --- | --- |
| $\varepsilon=ln(1+\varepsilon_{eng})$ | Eq. 12 |

# Regions of interest

Due to the low number of biopsies (n=12) multiple measurements were taken within the biopsies. Within the biopsies lamellar bone region were chosen as region of testing. 69 micropillars were milled using focused ion beam milling, while only 66 micropillar were successfully tested. Three micropillars failed during testing and had to be excluded (Reasons: There was a void inside the first pillar in biopsy H3 (probably an osteocyte inside); the second pillar was significantly weaker in biopsy OI type I 2 (no osteonal region) and the third pillar had to be aborted during testing due to a high drift of the loadcell in biopsy OI type I 3). Around the micropillar, three Raman spectra and nanoindentation were conducted (Sup. fig. 1 A). The final number of locations with Raman spectroscopy and nanoindentation was 66. In total, 198 (3x66) Raman measurements and 184 nanoindentations were successfully performed (14 indention failed due to contact problems). For both Raman spectroscopy and the nano indentation, the average value per location were computed (Sup. fig. 1 A). For some cases, multiple measurements were taken in within the same lamellar/ osteonal region (nested measurements, see sub fig 1 B)).

| 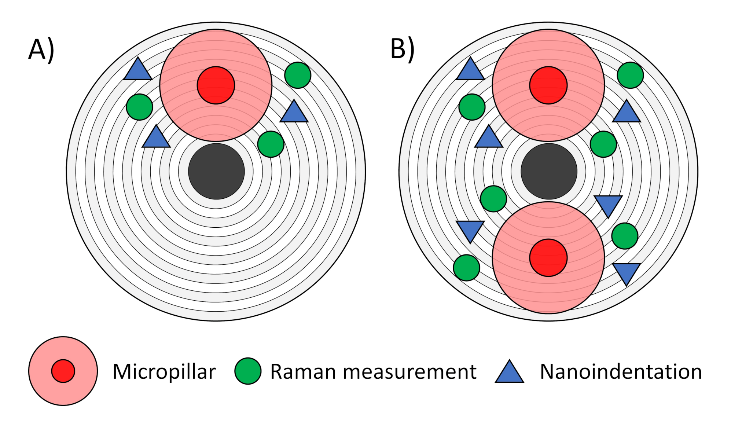 |
| --- |
| Sup fig 1:Scheme of region of interests (ROIs); Micropillars, Raman spectroscopy and Nanoindentation were conducted in lamellar bone. A) ROI for one micropillar and the corresponding mean value of three Raman spectras and Nanoindentations, B) ROI of additiona locatioen with the mean value of three Raman spectras and Nanoindentations, C) nested measurments within one osteonal region |

The following table reports the number of measurements taken per group, biopsy and osteonal region (see sup. tab. 1).

Sup. table 1: Overview of the fabricated micropillars and the number of measurements taken within the groups, biopsies and the osteonal regions. The number in parentheses in the last column represents the failed micropillars during testing

| Group | Sample ID | Osteonal region | Total number of fabricated micropillars | Total number of tested ROI |
| --- | --- | --- | --- | --- |
| Healthy control | H1 | Osteonal region 1 | 1 | 1 |
|  |  | Osteonal region 2 | 3 | 3 |
|  |  | **Total** | **4** | **4** |
|  | H2 | Osteonal region 1 | 3 | 3 |
|  |  | Osteonal region 2 | 3 | 3 |
|  |  | **Total** | **6** | **6** |
|  | H3 | Osteonal region 1 | 2 | 1 (-1) |
|  |  | Osteonal region 2 | 1 | 1 |
|  |  | **Total** | **3** | **2 (-1)** |
|  | H4 | Osteonal region 1 | 1 | 1 |
|  |  | Osteonal region 2 | 2 | 2 |
|  |  | Osteonal region 3 | 1 | 1 |
|  |  | **Total** | **4** | **4** |
|  | H5 | Osteonal region 1 | 1 | 1 |
|  |  | Osteonal region 2 | 1 | 1 |
|  |  | Osteonal region 3 | 1 | 1 |
|  |  | **Total** | **3** | **3** |
|  | H6 | Osteonal region 1 | 2 | 2 |
|  |  | Osteonal region 2 | 1 | 1 |
|  |  | Osteonal region 3 | 1 | 1 |
|  |  | Osteonal region 4 | 1 | 1 |
|  |  | **Total** | **5** | **5** |
|  | **Total** |  | **25** | **24 (-1)** |
| OI type I | OI type I 1 | Osteonal region 1 | 4 | 3 (-1) |
|  |  | Osteonal region 2 | 2 | 2 |
|  |  | **Total** | **6** | **5 (-1)** |
|  | OI type I 2 | Osteonal region 1 | 2 | 2 |
|  |  | Osteonal region 2 | 1 | 1 |
|  |  | Osteonal region 3 | 3 | 3 |
|  |  | Osteonal region 4 | 1 | 1 |
|  |  | **Total** | **7** | **7** |
|  | OI type I 3 | Osteonal region 1 | 2 | 1 (-1) |
|  |  | Osteonal region 2 | 1 | 1 |
|  |  | Osteonal region 3 | 2 | 2 |
|  |  | **Total** | **5** | **4 (-1)** |
|  | Total |  | **18** | **16 (-2)** |
| OI type IV | OI type IV 1 | Osteonal region 1 | 1 | 1 |
|  |  | Osteonal region 2 | 1 | 1 |
|  |  | Osteonal region 3 | 2 | 2 |
|  |  | Osteonal region 4 | 1 | 1 |
|  |  | Osteonal region 5 | 2 | 2 |
|  |  | **Total** | **7** | **7** |
|  | OI type IV 2 | Osteonal region 1 | 1 | 1 |
|  |  | Osteonal region 2 | 1 | 1 |
|  |  | Osteonal region 3 | 2 | 2 |
|  |  | **Total** | **4** | **4** |
|  | **Total** |  | **11** | **11** |
| OI type III | OI type III 1 | Osteonal region 1 | 3 | 3 |
|  |  | Osteonal region 2 | 2 | 2 |
|  |  | Osteonal region 3 | 1 | 1 |
|  |  | Osteonal region 4 | 1 | 1 |
|  |  | Osteonal region 5 | 1 | 1 |
|  |  | Osteonal region 6 | 5 | 5 |
|  |  | Osteonal region 7 | 2 | 2 |
|  |  | **Total** | **15** | **15** |
| **Total** |  |  | **69** | **66 (-3)** |

# Boxplots

Figure 2 visualize additional group comparison for hardness (A), hardness indentation ratio (B) and relative dissipated energy (C)


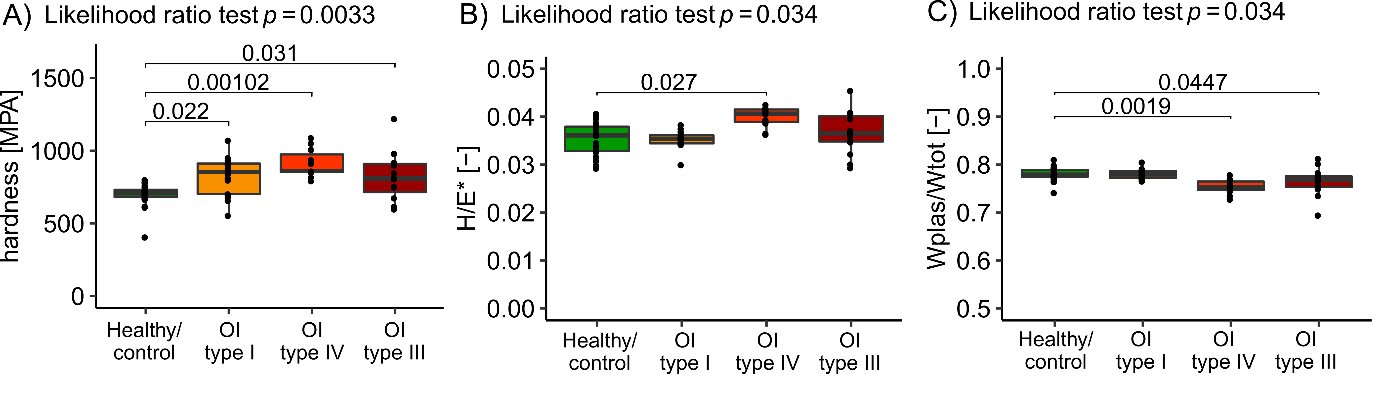


Sup. Fig 2: Comparison between groups of A) hardness, B) hardness-modulus ratio and C) relative dissipated energy

# Model to predict the influence of mineralization for hydrated bone

For wet bone, the mineralization may have a different influence on the mechanical properties. Therefore, a shear lag model, initially developed by Jäger and Frazl et al. (2000)^(2)^, was extended in Alizadeh et al. (2020)^(3)^ to describe the longitudinal Young's modulus of dry bone ECM. This model is extended here to wet bone ECM and the influence of mineral content is compared between the dry and wet cases.

**Definition and assumption of the model**

Following Alizadeh et al. (2020)^(3)^, the ECM is divided into a fibril and a surrounding extra-fibrillar matrix. The fibril is composed of intrafibrillar minerals and collagen. The extra-fibrillar matrix plays the role of a compliant ductile glue that is composed of extra-fibrillar mineral and non-collagen proteins. Water is distributed homogeneously by weight in the organic phases and appears therefore in both intra- and extra fibrillar space. The mass densities of the dry constituents and water are fixed. The ratio of intra- versus extra-fibrillar mineral content is fixed and assumed to be 0.6.

The two independent parameters of the developed shear lag model are 1) the water content and 2) the relative mineral to dry organic content.
The stiffnesses of the dry constituents are assumed to be constant, but the elastic moduli of the organic constituents are scaled with the swelling volume produced in the presence of water. The model consists of three nested shear lag models, one for the fibril, one for the extra-fibrillar matrix and one for the composition of the two.

**Results and Discussion**

For a mineral fraction of 60%, the model of dry bone delivers a Young's modulus of 22 GPa and predicts a reduction of 54% for wet bone, which is in line with the observations of the current study (~24 GPa) and the reduction of about 65% of yield stress measured by Schwiedrzik et al. (2015, Acta Biomaterialia) on wet micropillars in ovine bone (Young's modulus was not estimated but correlates generally strongly with yield stress).

In fact, Young's modulus increases monotonically with mineral content in both dry and fully wet conditions (Figure 3). However, our model indicates that the slope of this relationship is reduced in proportion to Young's modulus for fully wet bone. This suggests that the strong linear dependence of the elastic modulus with mineral to matrix ratio observed in dry bone does not disappear or does not become negative in wet bone. As a main limitation, this model does not account for biophysically/biochemically altered load transfer between the phases in the presence of water. Accordingly, despite the difficulty of obtaining fresh OI tissue, experimental verification on wet OI bone will become necessary to resolve this issue definitively.

Sup. Fig. 3: Youngs modulus of the fibril array (FA) versus mineral content for wet and dry bone predicted by the nested shear lag model.

# References

1. Zhang H, Schuster BE, Wei Q, Ramesh KT. The design of accurate micro-compression experiments. Scr. Mater. 2006;54(2):181–6.

2. Jäger I, Fratzl P. Mineralized collagen fibrils: A mechanical model with a staggered arrangement of mineral particles. Biophys. J. 2000;79(4):1737–46.

3. Alizadeh E, Dehestani M, Zysset P. An efficient two-scale 3D FE model of the bone fibril array: comparison of anisotropic elastic properties with analytical methods and micro-sample testing. Biomech. Model. Mechanobiol. [Internet]. Springer Berlin Heidelberg; 2020;(0123456789). Available from: https://doi.org/10.1007/s10237-020-01328-1
